# Development of the Inner City attitudinal assessment tool (ICAAT) for learners across Health care professions

**DOI:** 10.1186/s12913-020-5000-6

**Published:** 2020-03-06

**Authors:** Mark McKinney, Katherine E. Smith, Kathryn A. Dong, Oksana Babenko, Shelley Ross, Martina A. Kelly, Ginetta Salvalaggio

**Affiliations:** 1 Inner City Health and Wellness Program, Edmonton, AB Canada; 2 Department of Emergency Medicine, University of Ottawa, Ottawa, ON Canada; 3 Alberta Health Services, Edmonton, AB Canada; 4 Department of Emergency Medicine, University of Alberta, Edmonton, AB Canada; 5 Department of Family Medicine, University of Alberta, Edmonton, AB Canada; 6 Department of Family Medicine, University of Calgary Cumming School of Medicine, Calgary, AB Canada; 7 Department of Family Medicine, University of Alberta Faculty of Medicine & Dentistry, 610 University Terrace, Edmonton, AB T6G 2T4 Canada

**Keywords:** Vulnerable Populations, Underserved Populations, Marginalized Populations, Social Marginalization, Attitude of Health Personnel, Undergraduate Medical Education, Nursing Education

## Abstract

**Background:**

Many health professions learners report feeling uncomfortable and underprepared for professional interactions with inner city populations. These learners may hold preconceptions which affect therapeutic relationships and provision of care. Few tools exist to measure learner attitudes towards these populations. This article describes the development and validity evidence behind a new tool measuring health professions learner attitudes toward inner city populations.

**Methods:**

Tool development consisted of four phases: 1) Item identification and generation informed by a scoping review of the literature; 2) Item refinement involving a two stage modified Delphi process with a national multidisciplinary team (*n* = 8), followed by evaluation of readability and response process validity with a focus group of medical and nursing students (*n* = 13); 3) Pilot testing with a cohort of medical and nursing students; and 4) Analysis of psychometric properties through factor analysis and reliability.

**Results:**

A 36-item online version of the Inner City Attitudinal Assessment Tool (ICAAT) was completed by 214 of 1452 undergraduate students (67.7% from medicine; 32.3% from nursing; response rate 15%). The resulting tool consists of 24 items within a three-factor model – affective, behavioural, and cognitive. Reliability (internal consistency) values using Cronbach alpha were 0.87, 0.82, and 0.82 respectively. The reliability of the whole 24-item ICAAT was 0.90.

**Conclusions:**

The Inner City Attitudinal Assessment Tool (ICAAT) is a novel tool with evidence to support its use in assessing health care learners’ attitudes towards caring for inner city populations. This tool has potential to help guide curricula in inner city health.

## Background

Inner city populations are groups of marginalized individuals in urban settings who live with any combination of poverty, unstable housing, mental health issues, problematic substance use and involvement in survival sex or drug trade [1, 2]. The term ‘inner city’ remains common in Canada; however, phrases used to describe inner city populations in other settings may include ‘marginalized populations’, ‘vulnerable populations’, or ‘urban underserved populations’. [3] This is a difficult population to holistically define, in part due to the evolving nature of language and the social context in which terminology is used. Despite being a heterogeneous patient population, its members share similar unmet needs for care and past care experiences [2, 3].

Inner city populations have a high burden of illness and mortality but less access to effective primary care [4–6]. Providing health services to this population is often difficult because of mutual mistrust, population heterogeneity and the unique circumstances surrounding each individual. The provision of more holistic and evidence-based care to high-risk groups, which make up a higher proportion of frequent health-care users, is urgently needed [5, 7].

Despite regular opportunity for interaction with this population, learners may feel uncomfortable or underprepared for professional interactions with individual patients and their unique context and health care needs [8]. Learners may hold negative beliefs and/or attitudes which undermine the therapeutic relationship and may contribute to poor health outcomes in this group [9, 10]. Over the course of health professions training, some learners develop progressively more negative attitudes towards, and greater reluctance to work with, specific marginalized populations [1, 8]. Conversely, supported exposure to inner city patients and focused curricula can improve attitudes towards at-risk populations and increase the likelihood that learners will choose to work with these groups [11–13].

Measuring attitudes is one component of evaluating the impact of curricular interventions. Attitudes can be thought of as ‘a relatively enduring organization of beliefs, feelings, and behavioural tendencies towards socially significant objects, groups, events or symbols’, although many definitions have been proposed [14, 15]. In a commonly endorsed tripartite model, attitude is defined as a construct comprising affective, behavioural, and cognitive components [16, 17]. A tool measuring learner attitudes towards members of the inner city would assist in evaluating curricula designed to improve those attitudes. While a literature review identified a number of published tools examining attitudes toward specific subpopulations (e.g. populations defined solely by homelessness or single health conditions like mental health issues, substance use or HIV positive status) [18–26], no tool was sufficiently broad enough in context or language to apply to complex inner city populations that are encountered in generalist medical settings, such as in emergency or primary care [27]. Attempting to apply existing tools would be insufficient to capture the experience of a healthcare professional caring for a patient who presents with multiple issues resulting from a complex interplay of health and social concerns, rather than an isolated single health condition or social problem. A generalist lens supports the delivery of comprehensive, high quality health care to complex populations; assessing competency in this area requires an equally comprehensive, generalist stance.

The objective of this study was to develop and provide validity evidence for a tool to measure health care learner attitudes towards inner city populations.

## Methods

### Overview

**Fig. 1:**
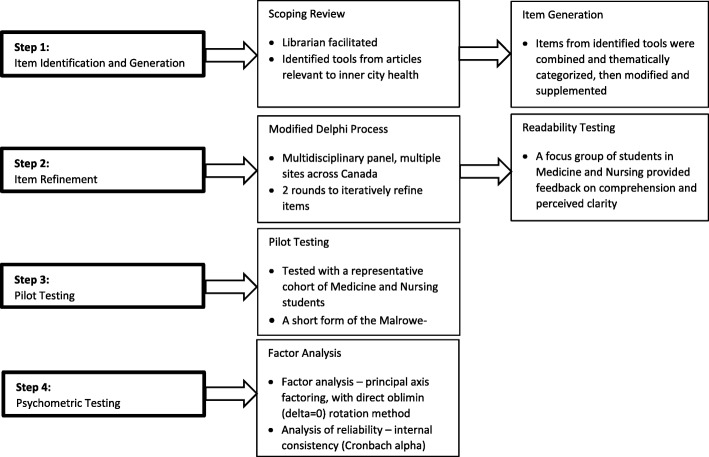
Overview of methodology

The methods outlined by Burns et al. were adopted for tool development (Fig. 1) [28]. The approach consisted of four phases:

1. Item identification and generation informed by a scoping review of the literature;
2. Item refinement involving a two-stage modified Delphi process with a national multidisciplinary team from Canada (*n* = 8), followed by evaluation of readability and response process validity with a focus group of medical and nursing students (*n* = 13);
3. Pilot testing with a larger cohort of medical and nursing students; and
4. Analysis of psychometric properties through factor analysis and analysis of reliability with the pilot test cohort of medical and nursing students (*n* = 214).

Approval for the study was obtained from local institution ethics boards: The Health Research Ethics Board – Health Panel at the University of Alberta, and the Conjoint Health Research Ethics Board at the University of Calgary.

### Item identification and generation

#### Identification of existing tools

**Fig. 2:**
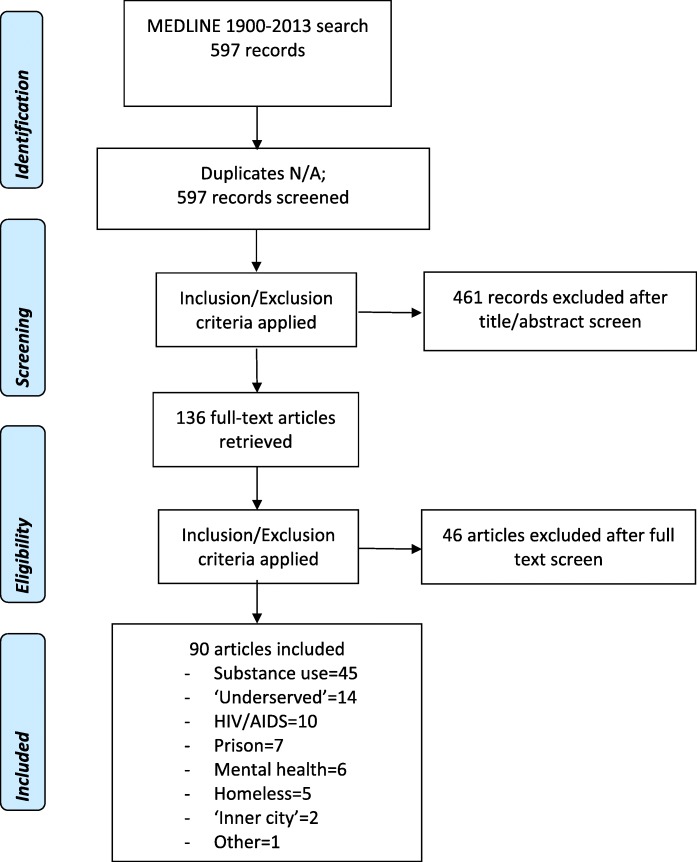
Study Selection and Results of Search for Existing Tools [31]

A librarian-facilitated search to identify existing tools, following methodology outlined by Arksey and O’Malley [29] and Levac [30] was performed to identify previously published tools for assessing health care learner attitudes toward inner city populations [27]. The search strategy (see Additional File 1.pdf) identified papers on inner city sub-populations (e.g. underserved populations, homelessness, addictions, etc.) and education across multiple health care disciplines and contexts. Articles were screened by 2 of 3 members of the research team and included articles were extracted for review (Fig. 2).

#### Preliminary item generation

Common attitudes relevant to delivering quality health care to inner city populations were mapped to conceptual themes as identified by the research team. Specific questions or statements (referred to as ‘items’) related to those themes were chosen from tools identified in the literature review [18–26, 32–34]. Items were drawn from the identified tools by a multidisciplinary team of experts to meet the following criteria:

1. Modifiable to apply to inner city populations as a whole;
2. Drawn from tools that had published validity evidence;
3. Publicly accessible and not from tools that were commercialized products;
4. Avoided duplication of concepts; and
5. Not exclusively assessing content knowledge.

Items were then adapted to apply to inner city populations. For example, the item ‘I often feel uncomfortable when working with drug users’ was reworded to ‘I often feel uncomfortable when interacting with patients from the inner city’. [18] New items were created by the research team if a topic was deemed important by the research team, but did not seem to be addressed by items in the available tools. For example, to address ‘empathy’ a new item ‘I avoid contact with people from the inner city when I am outside of a health care setting’ was created.

### Item refinement

#### Modified Delphi process

A modified Delphi method was used to incorporate expert opinion into item generation and refinement. Through a qualitative, iterative process, experts nominated and rated items. As this was a modified Delphi process, rather than seeking consensus, feedback was used to reach acceptable agreement.

The expert panel was chosen using convenience and word of mouth sampling. It included an outreach worker with lived inner city experience, clinicians (nurses, a nurse practitioner, and physicians), and researchers, from multiple Canadian provinces, who had experience with inner city health and/or educational assessment.

There were two rounds of expert feedback. Responses were collected and managed using REDCap electronic data capture tools hosted by the University of Alberta Women & Children’s Health Research Institute [35]. In the first round, experts rated the importance of each item on a labeled visual analogue scale (VAS) from 0 to 100, where 0 represented ‘Do not include’ and 100 represented ‘Critical to include’. Comment boxes for each item invited content and wording feedback. Experts were then asked to comment on the importance of each conceptual theme.

Items and themes were selected for the second round in an iterative, qualitative fashion whereby comments from the expert panel were taken into account and applied to rewrite items or item wording. Based on results from the first round of the Delphi process, low scoring items (< 65 received from two or more panel members) were removed or reworded, and remaining items were adapted to reflect comments from the panel. These items were categorized into new conceptual themes, and items that did not fit into a theme were removed. Retained items were expanded upon to evaluate alternative items with slight changes in wording or meaning.

In the second round, panel members were asked to score items on a four-point Likert scale (Essentially important, Important, Not important, Not relevant). The rationale for this change was that the VAS responses demonstrated a multimodal distribution (i.e. > 80 very good, 60–80 good, < 50 bad, < 10 very bad). A four-point scale offered better interpretability between panelist responses and provided a more definitive response. Comment boxes were again provided.

#### Readability testing in focus Group of Learners

A focus group of learners from health care professions evaluated remaining items for clarity and response process validity. Participants were recruited via email sent to University of Alberta medicine and nursing student listserves. Three research team members moderated the session. Participants reviewed items for conceptual clarity (‘Do these concepts make sense?’) and wording clarity (‘Can the wording be interpreted more than one way?’). Items were provided in random order on a paper copy for students to hand-write their feedback individually prior to group discussion. Written feedback was collected and field notes were made of the group discussion. The research team incorporated feedback from the focus group into the final text of items.

### Pilot testing

Pilot testing of the preliminary tool was conducted with a group of health professions learners. Participants included medical and nursing student cohorts from pre-clinical program years. Medical students were in the first half (first or second year) of their program, a period in which some longitudinal clinical experiences take place but most teaching occurs outside of the clinical setting. Nursing students were in the first two years of a four-year program, with recruitment occurring prior to any organized clinical experiences. These cohorts were chosen because they were of sufficient size to generate a large number of responses; whereas same-year cohorts from other local professional schools, such as midwifery or dentistry (fewer than 40 in each), were too small. Pre-clinical students were targeted to obtain validity evidence with a group of learners who had yet to have extensive clinical experience, providing relative homogeneity in their exposure to patients from inner city populations. Students were recruited via e-mail and in-class announcements, and were provided with an electronic link to the tool. A consent form was included in the email with a link to the REDCap survey software. The responses were anonymous; however, voluntary demographic information was collected, including age, gender, program, and year of study, for the purposes of sample characterization. There was no connection with learners’ academic programs regarding incentives or coercion to complete the study. Respondents could complete the survey in their own time over two months, after which the survey was closed. Responses were collected over two periods in 2015 and 2016 to reach the minimum sample size of 200 required for factor analysis of a < 40-item scale [36].

To examine social desirability bias, or the extent to which the respondent answers survey items in a socially desirable manner, a short form of the Malrowe-Crowne (MC) social desirability scale was included [37]. This scale has good psychometric properties and generalizability to many fields [37–40]. The items were incorporated in random order in the pilot test of the ICAAT items to evaluate for degree of social desirability bias in responses from the sample, and to determine if any items loaded more strongly with social desirability items rather than with ICAAT items.

The online questionnaire included 36 ICAAT items and 13 MC items. Participants indicated their level of agreement with each item using a six-point Likert-type scale (1–strongly disagree; 2–disagree; 3–somewhat disagree; 4–somewhat agree; 5–agree; 6–strongly agree).

### Psychometric testing - factor structure and reliability

SPSS 24.0 was used to analyze the data [41]. Factor analysis was performed to examine the internal structure of the items, using principal axis factoring for factor extraction and direct oblimin (delta = 0) rotation method, given the conceptual relatedness of the underlying concepts. Cattell scree test, Kaiser criterion (eigen-values greater than one), and conceptual meaningfulness of factors and respective items were used as criteria for factor and item retention [42–44]. Per recommended minimum cut-off for exploratory factor analysis, factor loadings of 0.40 for individual items were used in determining the complexity of resulting factors and for purposes of retaining the best fitting items in the final solution [45]. Reliabilities (internal consistency) were determined using Cronbach alpha.

## Results

### Item identification and generation

#### Identification of existing tools

Screening of 597 articles identified 90 attitudinal assessment tools that addressed components potentially relevant to inner city health (Fig. 2). No single tool applied to inner city populations as a whole. Of the identified tools, 11 contained items which met the target criteria (see Additional File 2.pdf).

#### Preliminary item generation

Through conceptual analysis, the research team assembled 54 relevant items (where seven items were entirely original, and the remaining 47 items were a result of rewording and removal of redundant items from existing tools) into seven themes that reflected attitudes toward inner city populations. Themes were chosen by researcher consensus and informed by existing literature, with the intent to provide initial organization as opposed to a final structure to the tool. These themes were initially labeled Role adequacy, Role legitimacy, Role support, Personal responsibility, Societal responsibility, Fear vs. comfort, and Non-stigmatization.

### Item refinement

#### Modified Delphi process

In round 1 of the modified Delphi process, the expert panel (*n* = 8) reviewed the included 54 items organized within these seven themes. Based on their responses, the items were refined and the conceptual themes were reorganized. In round 2, the expert panel was presented with 84 items. The increase in items reflected new items created to explore different wording choices. For example, panel members were asked their preference between the following sentences: ‘My profession should be able to refuse care to someone if they refuse to change their lifestyle’ and ‘I should be able to refuse care to someone who refuses to change their lifestyle’. Panel responses from the second round resulted in 45 items.

#### Readability testing in focus Group of Learners

Seven preclinical medical students and six undergraduate nursing students participated in the focus group and reduced the tool from 45 to 36 items. Most items underwent subtle wording changes reflective of student feedback. Students were sensitive to the negative connotations in some of the items and felt this influenced their opinion as they read through the list. This prompted modifications to ensure roughly equal numbers of positively and negatively connotative statements for each conceptual theme. For example, ‘It is not worth my time to provide care to someone from the inner city’ was modified by removing the word ‘not’. Some synonymous word choices were adopted as per student suggestions. For example, the word ‘futile’ was changed to ‘pointless’ to facilitate comprehension.

The resulting list consisted of 36 items within six conceptual themes (nine items within Professional responsibility, three within Stigma, six within Comfort, six within Futility, seven within Empathy, and five within Perceived competency). These 36 items formed the preliminary items used for pilot testing and later factor analysis.

### Pilot testing

Survey responses were received from 217 students, of which 214 completed the online tool (three began but did not finish). Demographic characteristics of respondents are provided in Table 1. All completed responses were included in factor analysis. Assuming all members of the cohort classes received the recruitment email, the total cohort size is 1452 (324 medical students from the University of Calgary; 487 medical students from the University of Alberta; and 641 nursing students from the University of Alberta), suggesting a 15% response rate.

**Table 1:** Demographic characteristics of pilot testing sample cohort (completed surveys, *n* = 214)

Characteristic		Frequency
Program	Medicine	147 (67.7%)
	Nursing	70 (32.3%)
Year of Study	1st	132 (60.8%)
	2nd	71 (32.7%)
	3rd	8 (3.7%)
	4th	6 (2.8%)
Gender	Female	155 (71.4%)
	Male	61 (28.1%)
	Other	1 (0.5%)
Age	19 or younger	27 (12.4%)
	20–24	127 (58.5%)
	25–29	39 (18.0%)
	30 or older	24 (11.1%)

### Psychometric testing – factor and structure reliability

The factor analysis results yielded evidence against social desirability bias in how the participants completed the tool. All MC social desirability items loaded on a separate factor and none of the ICAAT items loaded on this factor. Hence, the MC items were removed from later analysis.

Principal axis factoring indicated a three-factor model explaining 51.3% of the variance (prior to factor rotation). Twenty-four items were retained in the final form (Table 2). Factor 1 had eleven items loaded on it; Factor 2 had five items; and Factor 3 had eight items. These factors were named ‘Affective’, ‘Behavioural’, and ‘Cognitive’, respectively. Using Cronbach alpha, the reliabilities (internal consistency) of the resulting factors were 0.87, 0.82, and 0.82, respectively. The reliability of the whole 24-item ICAAT was 0.90.

**Table 2:** Factor loadings for the ICAAT items using principal axis factoring and direct oblimin (delta = 0) rotation (*n* = 214 students)

Items^a^	Factors		
	1	2	3
I feel uneasy when interacting with patients from the inner city.^RC^	**.700**	−.144	−.051
I feel uncomfortable when I talk to a patient from the inner city about their social circumstances.^RC^	**.688**	−.111	.183
I feel uneasy when I am in a room alone with someone from the inner city.^RC^	**.652**	−.063	−.064
I find it difficult to work with patients from the inner city because I have no way of relating to them.^RC^	**.643**	−.130	−.042
I avoid contact with people from the inner city when I am outside of a health care setting.^RC^	**.605**	−.040	−.045
I find it difficult to view things from the perspective of a patient from the inner city.^RC^	**.599**	−.241	.156
I am reluctant to talk to patients from the inner city about their social circumstances.^RC^	**.552**	−.148	−.061
People from the inner city are disruptive to health care staff and other patients.^RC^	**.501**	.203	−.143
People from the inner city overuse the health system and waste health care dollars.^RC^	**.459**	.201	−.292
I avoid contact with people from the inner city when I am in a health care setting.^RC^	**.452**	−.181	−.266
People from the inner city do not adequately value their own health.^RC^	**.430**	.247	−.230
I feel capable of communicating effectively with a patient from the inner city.	−.143	**.751**	.040
I feel I know enough about the health issues of inner city populations to provide care to a patient from the inner city.	.015	**.717**	.100
I feel that I know enough about the social determinants of health to provide care to a patient from the inner city.	−.068	**.650**	.006
I feel capable of facilitating trust with a patient from the inner city in a professional setting.	−.256	**.481**	.106
I feel capable of establishing a good working rapport with patients from the inner city.	−.351	**.476**	.053
Professionals in my discipline should advocate for the health of inner city populations.	.028	−.071	**.726**
It is my professional responsibility to provide care to underserved populations.	.071	.080	**.708**
It is worth my time to provide care to someone from the inner city.	−.166	.032	**.646**
My profession should be involved in providing care to underserved populations.	.094	.127	**.613**
Providing care to inner city populations is pointless. ^RC^	.124	.158	**.579**
A person from the inner city deserves hospital space and resources as much as any other patient.	−.056	−.004	**.578**
Professionals in my discipline should adapt how care is provided in order to meet the needs of patients from the inner city.	.017	.016	**.530**
Professionals in my discipline should address social determinants of health (such as unstable housing) when interacting with patients.	−.126	.067	**.478**

^a^ Items are listed by the order of the magnitude of the factor coefficient within each factor. Items were answered on a six-point Likert-type scale (1–strongly disagree; 6–strongly agree). Factor loadings greater than 0.40 are shown in bold.

^RC^ Indicates reverse coding.

Factor 1 is considered as a construct involving “Affective”

Factor 2 is considered as a construct involving “Behavioural”

Factor 3 is considered as a construct involving “Cognitive”

Bivariate correlations among three factors were < 0.48 in absolute value.

The three factors that emerged statistically are consistent with themes that assess different aspects of the ‘Attitude’ construct. The ‘Affective’ factor captures concepts of stigma, comfort during encounters with persons from the inner city, and empathy. The ‘Behavioural’ factor assesses self-reported abilities central to working with an inner city population, including communication skills, ability to facilitate trust and rapport, and knowledge of social inequities. The ‘Cognitive’ factor evaluates learner perspectives on the responsibilities of society, healthcare, and their profession, including advocacy for patients from the inner city, responsibility to provide care, and the importance of social determinants of health in caring for patients from the inner city.

## Discussion

The tool developed in this study, the Inner City Attitudinal Assessment Tool (ICAAT, Table 3), consists of 24 items within three conceptual themes (‘Affective’, ‘Behavioural’, and ‘Cognitive’). The validity evidence for the ICAAT was generated from a multidisciplinary, pre-clinical health care learner cohort. The factors identified in the ICAAT were noted to parallel previous research examining the construct of “Attitude”, notably the tripartite model [14–17]. The ICAAT can contribute to health sciences education, by offering a literature- and expert-informed tool to measure health professions learners’ attitudes about providing care to members of inner city populations.

**Table 3:** Inner City Attitude Assessment Tool (ICAAT). Participants are instructed to indicate their level of agreement with each item using a six-point Likert-type scale (1–strongly disagree; 2 – disagree; 3 – somewhat disagree; 4 – somewhat agree; 5 – agree; 6–strongly agree). The items are meant to appear in a random format. The following preamble may appear with the items: ‘This tool assesses attitudes towards inner city populations. Your responses will remain anonymous. Please answer the following as honestly as possible.’

Factor 1 – Affective	1. I feel uneasy when interacting with patients from the inner city. 2. I feel uncomfortable when I talk to a patient from the inner city about their social circumstances. 3. I feel uneasy when I am in a room alone with someone from the inner city. 4. I avoid contact with people from the inner city when I am outside of a health care setting. 5. I avoid contact with people from the inner city when I am in a health care setting. 6. I find it difficult to work with patients from the inner city because I have no way of relating to them. 7. I find it difficult to view things from the perspective of a patient from the inner city. 8. I am reluctant to talk to patients from the inner city about their social circumstances. 9. People from the inner city are disruptive to health care staff and other patients. 10. People from the inner city do not adequately value their own health. 11. People from the inner city overuse the health system and waste health care dollars.
Factor 2 – Behavioural	1. I feel I know enough about the health issues of inner city populations to provide care to a patient from the inner city. 2. I feel that I know enough about the social determinants of health to provide care to a patient from the inner city. 3. I feel capable of establishing a good working rapport with patients from the inner city. 4. I feel capable of communicating effectively with a patient from the inner city. 5. I feel capable of facilitating trust with a patient from the inner city in a professional setting.
Factor 3 – Cognitive	1. It is my professional responsibility to provide care to underserved populations. 2. My profession should be involved in providing care to underserved populations. 3. Professionals in my discipline should address social determinants of health (such as unstable housing) when interacting with patients. 4. Professionals in my discipline should advocate for the health of inner city populations. 5. Professionals in my discipline should adapt how care is provided in order to meet the needs of patients from the inner city. 6. A person from the inner city deserves hospital space and resources as much as any other patient. 7. It is worth my time to provide care to someone from the inner city. 8. Providing care to inner city populations is pointless.

Inner city populations have disproportionate health care needs for their population size [4–6], yet are also a group that can be challenging to treat for a variety of reasons [5, 7]. The current model for health care is not effective for inner city populations whether looked at from a patient outcomes perspective, or a system cost perspective. A shift in training models is urgently needed. The common pattern of health care use by people within an inner city context is that of higher acuity on presentation, higher medical complexity, lower preventative health uptake, and higher health costs [46, 47]. The likelihood of poor outcomes for these patients is exacerbated when they are seen by staff who may have little to no training in managing health inequities resulting from social determinants of health [48]. Increasing evidence confirms the failure of crisis-oriented care delivery and the value of social determinants of health and relationship-centred care [49, 50], highlighting the need for effective training programs and curricula to expose health professions trainees to these contextual influences on health.

A shift in training is needed to ensure that inner city populations get appropriate care, and to improve health care outcomes for this population. Unfortunately, health care educators and role models may sometimes hold negative attitudes towards patients in crisis who are experiencing the impact of adverse social circumstances, past trauma, and untreated medical conditions; these negative attitudes may be modeled for learners, whose attitudes are also seen to worsen over the course of training [8, 51–53]. Any training program designed to address attitudinal competencies must be accompanied by ongoing program evaluation to monitor whether the programs are having the intended effect of positive changes in attitudes. The ICAAT is a tool that could assist in the evaluation of these programs through measuring the self-reported attitudes of the learners being trained. This could take the form of a before-and-after analysis of an educational intervention, or a pooled comparison of groups.

In recent years, health sciences education programs have been shifting to competency-based approaches. Several competency frameworks have been developed in response to this shift, such as the CanMEDS roles in Canada [54], the Accreditation Council of Graduate Medical Education competencies in the United States [55], and the Royal College of General Practitioners competencies in the United Kingdom [56]. These frameworks all emphasize the need for health professional learners to demonstrate competencies beyond medical knowledge. However, addressing non-clinical aspects of health care is relatively more difficult than addressing knowledge-based domains [57]. The ICAAT is structured to address attitudes toward inner city populations, and can potentially be used in learner self-assessment.

A strength of the ICAAT is that it was designed for use with more than one health care discipline, reflecting the multidisciplinary environment that characterizes our current health system; as such, the development process included input from representatives of different disciplines. The research team included medical and nursing professionals; the expert panel included representatives of medicine, nursing, social work, and community members with lived inner city experience; and the readability and pilot testing involved medicine and nursing students. By having more than one discipline represented in the development of the tool, there is greater likelihood that the final tool is useable in cohorts of learners in several health care disciplines.

Through use of items from a short form of the Marlow-Crowne social desirability scale [38], tendency of responses primarily due to social desirability bias was evaluated. Results from factor analysis yielded evidence against social desirability bias in the responses. This is a reassuring finding regarding the extent to which learners might provide answers to appear socially appropriate as opposed to answers representing their true attitudes. However, these results are taken in the context of anonymity of the respondents.

### Limitations

Some may find the term ‘inner city’ to be inadequate to define the population, or even pejorative in its meaning. Language evolves as the conceptual understanding of phrases changes, as does the social context in which they are used. Recognizing this challenge, the chosen phrasing is common and remains acceptable within the current Canadian context. Indeed, a number of Canadian health care services and related academic initiatives specifically reference inner city populations within their mandate. The term ‘inner city’ appeared to be sufficiently understood by the expert panel and students involved in readability testing, and the ICAAT concept was warmly received. However, this term might not be equally understood or accepted in all contexts in which the ICAAT might be employed. Where the term ‘inner city’ is less common, the ICAAT may perform differently, and educators might consider gathering validity evidence, with or without modified terminology, prior to widespread use in such settings.

It is beyond the scope of the ICAAT to assist with broader case finding for marginalizing circumstances. Inner city residence does not necessarily imply marginalization, nor are marginalizing conditions constrained to inner city locations. However, marginalizing conditions can congregate in specific urban settings, and students working in these communities should be mindful of this prevalence and poised to provide appropriate care. Moreover, exposure to inner city learning experiences, with the ICAAT as a means of self-reflection, will better equip students to address marginalization in a variety of settings [58].

Educators might worry that the use of instruments assessing attitudes will introduce or solidify implicit learner biases against inner city populations. Although we did not perform longitudinal assessment when gathering our validity evidence to refute this possibility, we did not elicit any concerns about a potential Hawthorne effect from participating student cohorts, medical and nursing school personnel, or Delphi panelists. If paired with targeted curriculum that acknowledges and challenges implicit biases, the ICAAT is unlikely to override that learning experience or have a net negative effect.

Although the connection is expected conceptually, a direct connection between attitudes addressed in the ICAAT with real-life practice and patient outcomes has not been made. We would expect that students who score higher on measures of positive attitudes towards inner city patients would have greater therapeutic alliance and presumably better patient outcomes, but one of the limitations of educational assessment tools is the difficulty in making explicit links to changes in future practice and patient outcomes. It has previously been shown that greater scores on measures of empathy, for example, can be linked to patient outcomes [59]. Notably, several items in the ICAAT address empathy, particularly in the first factor of ‘Affective’. Future research to examine the connection between attitudes measured by the ICAAT and real-world practice is warranted.

An important next step in tool development would be confirmatory factor analysis (CFA) in a larger representative cohort. CFA was not pursued because of the feasibility limitations of recruiting a large enough cohort. In the form described here, the ICAAT can be considered at an intermediate point on a continuum of tool development.

The modified Delphi process described herein was not a strict Delphi process in that it did not seek uniform consensus, but rather incorporated feedback from the experts. True consensus would not be feasible because panel members varied considerably with respect to specific expertise and discipline.

### Future work

The ICAAT will require testing with new cohorts to gather additional validity evidence. This includes testing with more experienced clinicians, such as medical residents and practicing nurses, as well as with other health care-oriented disciplines, such as social workers and pharmacists. Further, knowledge translation activities are underway to encourage uptake of the ICAAT in undergraduate clinical settings; this ‘real-world’ use of the ICAAT will provide additional feedback and the opportunity to refine the tool as needed.

## Conclusions

The 24-item ICAAT assesses health care learners across three conceptual themes relevant to inner city care, which have been designated ‘Affective’, ‘Behavioural’, and ‘Cognitive’. It has been developed through a literature and expert-informed process, with validity evidence from testing with a clinical health care learner cohort. This multidisciplinary tool can be used to promote learner reflection on attitudes and refine curricula in inner city health, with the ultimate goal of improving health care professional attitudes and care provision.

## Supplementary information

**Additional file 1.** Search strategy to identify tools used to measure attitudes to inner city populations.

**Additional file 2.** List of tools selected for preliminary item generation.

## Abbreviations

CFA: Confirmatory Factor Analysis
HIV: Human Immunodeficiency Virus
ICAAT: Inner City Attitudinal Assessment Tool
MC: Malrowe-Crowne
VAS: Visual Analogue Scale
