# Supplementary material for: Development of the Inner City attitudinal assessment tool (ICAAT) for learners across Health care professions
Source: BMC Health Serv Res. 2020 Mar 6;20:174. doi: 10.1186/s12913-020-5000-6 (PMC7059309; doi:10.1186/s12913-020-5000-6)
Supplement: Supplementary file 1 — Additional file 1. Search strategy to identify tools used to measure attitudes to inner city populations. [file 12913_2020_5000_MOESM1_ESM.pdf]

Additional File 1: Search strategy to identify tools used to measure attitudes to inner city populations.

(1) exp Homeless Persons/ or exp "Transients and Migrants"/ or ((vulnerable or migrant or transient*) adj2 (people or person* or individual* or child* or youth* or population* or worker* or men or women or man or woman)).mp. or (street adj2 (people or person* or individual* or youth* or population* or child* or men or women or man or woman)).mp. or ("lack of housing" or substandard housing or unstably housed or underhoused or squatter* or homeless*.mp. or homeless* or vagrant* or indigent).mp. or (marginal* adj2 (population* or people* or group* or hous*)).mp.

(2) exp poverty area/ or (slum or slums or ghetto* or barrio or barrios or inner city or downtown core or urban core or city core or skid row).mp. [mp=title, abstract, original title, name of substance word, subject heading word, keyword heading word, protocol supplementary concept, rare disease supplementary concept, unique identifier]

(3) exp Prisoners/ or prisoner*.mp. or inmate*.mp. [mp=title, abstract, original title, name of substance word, subject heading word, keyword heading word, protocol supplementary concept, rare disease supplementary concept, unique identifier]

(4) drug users/

(5) poverty/ or poverty.mp. or prostitut*.mp. or prostitution/ [mp=title, abstract, original title, name of substance word, subject heading word, keyword heading word, protocol supplementary concept, rare disease supplementary concept, unique identifier]

(6) (low* adj7 ((socioeconomic* or socio economic*) adj1 (status or class or position*))).ti.

(7) substance-related disorders/ or alcohol-related disorders/ or alcohol-induced disorders/

or alcoholic intoxication/ or alcoholism/ or binge drinking/ or amphetamine-related disorders/ or cocaine-related disorders/ or drug overdose/ or inhalant abuse/ or marijuana abuse/ or exp opioid-related disorders/ or psychoses, substance-induced/ or substance abuse, intravenous/ or substance withdrawal syndrome/ or exp alcohol withdrawal delirium/

(8) exp Homeless Persons/ or exp "Transients and Migrants"/ or ((vulnerable or migrant or transient*) adj2 (people or person* or individual* or child* or youth* or population* or worker* or men or women or man or woman)).mp. or (street adj2 (people or person* or individual* or youth* or population* or child* or men or women or man or woman)).mp. or ("lack of housing" or substandard housing or unstably housed or underhoused or squatter* or homeless*.mp. or homeless* or vagrant* or indigent).mp. or (marginal* adj2 (population* or people* or group* or hous*)).mp.

(9) exp poverty area/ or (slum or slums or ghetto* or barrio or barrios or inner city or downtown core or urban core or city core or skid row).mp. [mp=title, abstract, original title, name of substance word, subject heading word, keyword heading word, protocol supplementary concept, rare disease supplementary concept, unique identifier]

(10) exp Prisoners/ or prisoner*.mp. or inmate*.mp. [mp=title, abstract, original title, name of substance word, subject heading word, keyword heading word, protocol supplementary concept, rare disease supplementary concept, unique identifier]

(11) drug users/

(12) poverty/ or poverty.mp. or prostitut*.mp. or prostitution/ [mp=title, abstract, original title, name of substance word, subject heading word, keyword heading word, protocol

supplementary concept, rare disease supplementary concept, unique identifier]

(13) (low* adj7 ((socioeconomic* or socio economic*) adj1 (status or class or position*))).ti.

(14) substance-related disorders/ or alcohol-related disorders/ or alcohol-induced disorders/ or alcoholic intoxication/ or alcoholism/ or binge drinking/ or amphetamine-related disorders/ or cocaine-related disorders/ or drug overdose/ or inhalant abuse/ or marijuana abuse/ or exp opioid-related disorders/ or psychoses, substance-induced/ or substance abuse, intravenous/ or substance withdrawal syndrome/ or exp alcohol withdrawal delirium/

(15) exp Homeless Persons/ or exp "Transients and Migrants"/ or ((vulnerable or migrant or transient*) adj2 (people or person* or individual* or child* or youth* or population* or worker* or men or women or man or woman)).mp. or (street adj2 (people or person* or individual* or youth* or population* or child* or men or women or man or woman)).mp. or ("lack of housing" or substandard housing or unstably housed or underhoused or squatter* or homeless*.mp. or homeless* or vagrant* or indigent).mp. or (marginal* adj2 (population* or people* or group* or hous*)).mp.

(16) exp poverty area/ or (slum or slums or ghetto* or barrio or barrios or inner city or downtown core or urban core or city core or skid row).mp. [mp=title, abstract, original title, name of substance word, subject heading word, keyword heading word, protocol supplementary concept, rare disease supplementary concept, unique identifier]

(17) exp Prisoners/ or prisoner*.mp. or inmate*.mp. [mp=title, abstract, original title, name of substance word, subject heading word, keyword heading word, protocol supplementary concept, rare disease supplementary concept, unique identifier]

(18) drug users/

(19) poverty/ or poverty.mp. or prostitut*.mp. or prostitution/ [mp=title, abstract, original title, name of substance word, subject heading word, keyword heading word, protocol supplementary concept, rare disease supplementary concept, unique identifier]

(20) (low* adj7 ((socioeconomic* or socio economic*) adj1 (status or class or position*))).ti.

(21) substance-related disorders/ or alcohol-related disorders/ or alcohol-induced disorders/ or alcoholic intoxication/ or alcoholism/ or binge drinking/ or amphetamine-related disorders/ or cocaine-related disorders/ or drug overdose/ or inhalant abuse/ or marijuana abuse/ or exp opioid-related disorders/ or psychoses, substance-induced/ or substance abuse, intravenous/ or substance withdrawal syndrome/ or exp alcohol withdrawal delirium/

(22) (substance abuse* or substance misuse* or substance dependence or substance use*).ti,ab.

(23) (drug abuse* or drug addict* or drug use* or drug dependence or idu).ti.

(24) (alcoholic* or alcoholism* or alcohol use* or alcohol abuse* or alcohol misuse*).ti.

(25) (heroin use* or heroin dependence or heroin addict*).ti.

(26) (cocaine use* or cocaine dependence or cocaine addict*).ti.

(27) (illicit drug use* or intravenous drug use* or iv drug use* or parenteral drug use* or injection drug use*).ti.

(28) 18 or 19 or 20 or 21 or 22 or 23 or 24 or 25 or 26 or 27 or 28 or 29 or 30

(29) (attitud* or learner comfort).ti.

(30) ((attitud* or learner comfort) adj5 (assess* or test* or evaluate* or questionnaire* or

tool*)).tw.

(31) exp "Attitude of Health Personnel"/ or attitudes of health personnel.mp.

(32) 32 or 33 or 34

(33) 31 and 35

(34) exp emergency responders/ or health personnel/ or exp allied health personnel/ or exp dental staff/ or exp dentists/ or faculty, dental/ or faculty, medical/ or faculty, nursing/ or health educators/ or exp medical staff/ or exp nurses/ or exp nursing staff/ or exp personnel, hospital/ or pharmacists/ or exp physicians/ or exp students, health occupations/

(35) (social worker* or sociologist* or case worker* or counsellor* or psychologist* or police).tw.

(36) 37 or 38

(37) 36 and 39
