# Supplementary material for: Development of the Inner City attitudinal assessment tool (ICAAT) for learners across Health care professions
Source: BMC Health Serv Res. 2020 Mar 6;20:174. doi: 10.1186/s12913-020-5000-6 (PMC7059309; doi:10.1186/s12913-020-5000-6)
Supplement: Supplementary file 2 — Additional file 2. List of tools selected for preliminary item generation. [file 12913_2020_5000_MOESM2_ESM.pdf]

Additional File 2: List of tools selected for preliminary item generation

1. Watson H., Maclaren W. & Kerr S. (2006) Staff attitudes towards working with drug users: development of the Drug Problems Perceptions Questionnaire. Society for the Study of Addiction 102, 206–215.

2. DS Buck, FM Montiero, S Kneuper, D Rochon, D Clark, A Melillo & RJ Volk. BMC Medical Education 2005, 5:2. Design and validation of the Health Professionals' Attitudes Toward the Homeless Inventory (HPATHI).

3. M Habibian, L Elizondo & R Mulligan. Dental Students' Attitudes Toward Homeless People While Providing Oral Health Care. Journal of Dental Education 2010. Volume 74, Number 11, 1190-1196.

4. Brett Williams, Ted Brown, Malcolm Boyle, Vanessa Webb. The Medical Condition Regard Scale (MCRS): An examination of its factor structure using paramedic students. Nurse Education Today 33 (2013) 938–943.

5. Lai-Chu See, Yu-Ming Shena, Chia-Ling Chena,b, Tsuei-Mi Huangc, Yi-Hua Huanga,b, Hui-Chun Huangc and Sheue-Rong Linc. Professional attitude of health care workers toward serving HIV/AIDS patients and drug users: questionnaire design and evaluation of reliability and validity. AIDS Care Vol. 23, No. 11, November 2011, 1448-1455.

6. Miklos Zrinyi and Zoltan Balogh. STUDENT NURSE ATTITUDES TOWARDS HOMELESS CLIENTS: A CHALLENGE FOR EDUCATION AND PRACTICE. Nursing Ethics 2004 11 (4). 10.1191/0969733004ne707oa.

7. T Brown, B Williams, M Boyle, A Molloy, L McKenna, L Molloy & B Lewis. Levels of Empathy in Undergraduate Occupational Therapy Students. Occup. Ther. Int. 17 (2010) 135–141.

8. Marcus A. The alcoholism questionnaire: administration, scoring and interpretation. Toronto: Add Res Found; 1980.

9. Seaman J, Mannello T. Nurses' attitudes towards alcohol and alcoholism - The Seaman-Mannello scale. Arlington, VA: National Institute on Alcohol Abuse and Alcoholism. 1978

10. Tolor A, Tamerin JS. The attitudes instrument: a measure of attitudes towards alcoholics and the nature and causes of alcoholism. B J Add 1975; 70, 223-231 4. Ferneau EW, Morton EL. Nursing personnel and alcoholism. Nurs Res 1968;17:174-177.

11. Sandra Pillon, Ronaldo Laranjeira, John Dunn. Nurses' attitudes towards alcoholism: factor analysis of three commonly used scales. 1998. São Paulo Medical Journal/RPM 116(2): 1661-1666.
